# Effect of herd size on subclinical infection of swine in Vietnam with influenza A viruses

**DOI:** 10.1186/s12917-016-0844-z

**Published:** 2016-10-10

**Authors:** Nobuhiro Takemae, Yugo Shobugawa, Phuong Thanh Nguyen, Tung Nguyen, Tien Ngoc Nguyen, Thanh Long To, Phuong Duy Thai, Tho Dang Nguyen, Duy Thanh Nguyen, Dung Kim Nguyen, Hoa Thi Do, Thi Quynh Anh Le, Phan Truong Hua, Hung Van Vo, Diep Thi Nguyen, Dang Hoang Nguyen, Yuko Uchida, Reiko Saito, Takehiko Saito

**Affiliations:** 1 Influenza and Prion Diseases Research Center, National Institute of Animal Health, NARO, Ibaraki, Japan; 2 Thailand–Japan Zoonotic Diseases Collaboration Center, Bangkok, Thailand; 3 Division of International Health, Graduate School of Medical and Dental Sciences, Niigata University, Niigata, Japan; 4 Department of Animal Health, Center for Veterinary Diagnostics, Regional Animal Health Office No. 6, Ho Chi Minh City, Vietnam; 5 Department of Animal Health, Epidemiology Division, Hanoi, Vietnam; 6 Department of Animal Health, National Centre for Veterinary Diagnostics, Hanoi, Vietnam; 7 United Graduate School of Veterinary Sciences, Gifu University, Gifu, Japan

**Keywords:** Active surveillance, Influenza A virus, Pig, Pig farm, Vietnam

## Abstract

**Background:**

Influenza A viruses of swine (IAV-S) cause acute and subclinical respiratory disease. To increase our understanding of the etiology of the subclinical form and thus help prevent the persistence of IAV-S in pig populations, we conducted active virologic surveillance in Vietnam, the second-largest pig-producing country in Asia, from February 2010 to December 2013.

**Results:**

From a total of 7034 nasal swabs collected from clinically healthy pigs at 250 farms and 10 slaughterhouses, we isolated 172 IAV-S from swine at the weaning and early-fattening stages. The isolation rate of IAV-S was significantly higher among pigs aged 3 weeks to 4.5 months than in older and younger animals. IAV-S were isolated from 16 large, corporate farms and 6 family-operated farms from among the 250 farms evaluated. Multivariate logistic regression analysis revealed that “having more than 1,000 pigs” was the most influential risk factor for IAV-S positivity. Farms affected by reassortant IAV-S had significantly larger pig populations than did those where A(H1N1)pdm09 viruses were isolated, thus suggesting that large, corporate farms serve as sites of reassortment events.

**Conclusions:**

We demonstrate the asymptomatic circulation of IAV-S in the Vietnamese pig population. Raising a large number of pigs on a farm has the strongest impact on the incidence of subclinical IAV-S infection. Given that only some of the corporate farms surveyed were IAV-S positive, further active monitoring is necessary to identify additional risk factors important in subclinical infection of pigs with IAV-S in Vietnam.

**Electronic supplementary material:**

The online version of this article (doi:10.1186/s12917-016-0844-z) contains supplementary material, which is available to authorized users.

## Background

Influenza A viruses of swine (IAV-S) are important infectious agents that contribute to porcine respiratory disease complex [1–4]. Pigs infected with IAV-S typically demonstrate depression, anorexia, and labored abdominal breathing [5], and IAV-S frequently are isolated from pigs showing acute clinical signs [3]. The high morbidity rate associated with IAV-S, which reaches approximately 100 % among pigs kept together in the same pig pen or farm, causes heavy economic losses in the pig industry because of high medication costs and decreased weight gain [6]. In addition, our previous active surveillance of IAV-S in clinically healthy pigs in Thailand revealed that subclinical infection frequently affects pigs in the weaning and early-fattening stages on farrow-to-finish pig farms [7]. This finding suggests that IAV-S exist either in a subclinical form or in an epizootic form that causes the typical respiratory syndrome in pigs [8–11]. However, the dynamics of subclinical IAV-S in pig populations are not yet well understood, largely because most IAV-S surveillance is conducted passively—that is, by using samples submitted to a laboratory after outbreaks of respiratory disease.

According to serosurveillance data from Canada [12], seroprevalence for the H1N1 subtype was 61.1 % among 65 sow herds and 24.3 % among 72 finisher herds. In addition, an estimated 49.8 % of the finisher herds in the United States are infected with IAV-S [13]. Serologic studies on IAV-S in Canada [12], Belgium [1], France [4], Spain [14, 15], Malaysia [16], and England [17] have found that “high number of pigs per farm or pen”, followed by “importation or purchase of pigs” and “proximity of the farm to other pig farms”, are the key risk factors for increased rates of seropositivity against IAV-S. In addition, “way of the moving pigs on the farms” and “lack of all-in–all-out management” have been suggested as risk factors for H1N1 and H1N2 IAV-S, respectively [4]. Despite these studies, how IAV-S persist in pig populations during the intervals between disease outbreaks remains unclear, because serosurveillance cannot reveal the dynamics of infection with either subclinical or epizootic IAV-S.

IAV-S in Vietnam have not been studied thoroughly, even though Vietnam is considered a high-priority target country for improvements in IAV-S monitoring, given that its pig and poultry populations are large [18]. Vietnam was the second-largest producer of pork in Asia in 2012 [19], and about 85 % of the country’s total pig production comes from smallholders rearing fewer than 20 sows [20, 21]. In addition, these smallholders often concurrently raise multiple types of livestock, including pigs and poultry—a practice considered to support the interspecies transmission of influenza viruses as well as genetic reassortment between avian and mammalian influenza viruses [22]. Furthermore, large-scale production by commercial producers rose from 2 % to 17 % of the total pig product in the 2000s [21, 23], resulting in an increase of about 7 million pigs in Vietnam [19]; thus, industrialized production is gradually replacing traditional, small-farm production.

We have been monitoring Vietnamese IAV-S at the various types of pig holdings, including large- and small-scale farms and slaughterhouses, since February 2010 and have reported subclinical infections with novel reassortant H3N2 and H1N2 IAV-S and A(H1N1)pdm09 viruses in Vietnamese pigs from 2010 to 2011 [24, 25]. The reassortant H3N2 and H1N2 IAV-S affected corporate, large-scale farms, whereas the A(H1N1)pdm09 viruses were present on both large-scale farms and family-operated small farms. Overall, most of the farms that we investigated earlier had neither of these viruses. These findings prompted us to consider that management procedures might affect the incidence of subclinical IAV-S infection on pig farms in Vietnam. In addition, we surmised that risk factors for subclinical infection might differ between the enzootic strains in pig populations and the A(H1N1)pdm09 viruses, which were recently reintroduced into pig populations from humans [26]. To answer these questions, we collected information related to pig husbandry methods, including the number of pigs, biosecurity level, and type of operation, through interviews with farmers when we collected samples between February 2010 and December 2013 for virus isolation. We applied univariate and logistic regression analysis to the husbandry and virologic surveillance data to identify factors involved in the enzooticity of IAV-S in Vietnamese pigs.

## Methods

### Sample collection

We collected nasal swabs 17 times in total from pig farms in northern and southern Vietnam from February 2010 to December 2013. Nasal swab samples were collected twice or three times in a year during the period. We collected a total of 6659 nasal-swab samples from clinically healthy pigs on a total of 250 pig farms located in 10 provinces where pig farming was prevalent, comprising 3 provinces (Bac Ninh, Hanoi, and Nam Dinh) in the northern region and 7 provinces (Ba Ria Vung Tau, Binh Duong, Dong Nai, Long An, Ho Chi Minh, Soc Trang, and Tien Giang) in the southern region. Farms were recruited to include a variety of farms differing in total number of pigs reared and operation type to reflect the whole situation of Vietnamese pig industry. Owners’ consent was obtained upon enrolment. Previous history of influenza or respiratory disease was not considered as a condition for the enrollment. The first five collection events occurred during or before February 2011; nasal swabs were collected from groups of suckling pigs, weanling pigs, fattening pigs, mature sows, and adult boars. During the remaining twelve samplings, we sampled weanling and fattening pigs only. Of the 250 farms, 55 were visited more than once (2 to 6 times) and during different periods. We collected 14 to 30 nasal swabs from each farm at each visit, thus allowing us to detect at least one infected pig when the disease prevalence was 7.5 % to 20 % at 95 % confidence [27]. If a small farm had fewer than 14 pigs, nasal swabs were collected from all available pigs.

During December 2010, we visited 10 slaughterhouses located in Ho Chi Minh, Binh Duong, and Long An provinces and collected 375 nasal swabs in total. At each slaughterhouse we collected nasal swabs from 20 to 75 finishing pigs (age, approximately 6 months); samples were collected from as many pigs as possible at each slaughterhouse at each sampling time. In the same way as for the samples taken from pig farms, all of the samples from slaughterhouse pigs were collected from animals that lacked clinically evident respiratory disease.

Ethical approval was not required for this study, because taking a nasal swab does not cause pain and lasting harm to a pig. Informed consent was obtained from all participating pig farm/slaughter house owners.

### Virus isolation and characterization

Nasal swab samples were collected from pigs by using flocked swabs with a plastic handle (Ex Swab 001, Denka Seiken, Tokyo, Japan). The swab was inserted 7 to 10 cm from the external naris, and the nasopharynx was rubbed several times with the swab. Each sample-bearing swab was placed immediately into a 15-ml tube containing 2 ml transport medium (MEM containing penicillin [1000 unit/ml], streptomycin [1000 μg/ml], Fungizone [25 μg/ml] (Thermo Fisher Scientific, Waltham, MA, USA), 0.01 M HEPES, and 0.5 % bovine serum albumin) and kept at 4 °C. After centrifugation at 1400 × *g* for 5 min, the supernatant fluid was aliquoted and kept at −80 °C until use. Pooled supernatant (2 to 5 nasal swabs per pool) was screened by real-time PCR using SYBR Premix Ex Taq (Takara Bio, Shiga, Japan) with primers targeting the M gene of influenza A viruses, as previously described [24]. When a pooled sample was positive by PCR, the nasal swabs represented in the pooled samples were individually passed over a 0.45-μm pore-size filter (Millipore, Billerica, MA, USA) and the filtered materials was inoculated into floating MDCK cells. To prepare floating MDCK cells, monolayers of MDCK cells were washed 3 times with sterile PBS to eliminate residual fetal calf serum; 0.05 % trypsin–EDTA solution (Thermo Fisher Scientific) was then added and the trypsinized cells were collected into maintenance MEM containing penicillin (100 units/ml), streptomycin (100 μg/ml), Fungizone (2.5 μg/ml), gentamicin (100 μg/ml), and 0.4 % bovine serum albumin. After centrifugation at 400 × *g* for 5 min, the cells were resuspended at a concentration of 1.0 × 10^6^ cells/ml in maintenance MEM containing 5.0 μg/ml TPCK–trypsin (Thermo Fisher Scientific), and 1.6 ml was dispensed into each Nunc cell-culture tube (flat surface area 5.5 cm^2^) (Thermo Fisher Scientific). A 160-μl aliquot of the original nasal swab was inoculated into the resuspended MDCK cells and incubated for 4 days at 37 °C in 5 % CO_2_. When viruses were not isolated from the PCR-positive swab using floating MDCK cells, the original materials were inoculated into embryonated chicken eggs and/or primary cultures of porcine alveolar epithelial cells as previously described [25, 28]. Hemagglutination (HA) activity was tested by using 0.55 % red blood cells from guinea pigs or chickens as previously described [28]. If HA activity was not observed after the first passage, the supernatant fluids collected were inoculated into each substrate once more.

Nucleotide sequences of the viruses isolated were determined by direct sequencing of PCR products as previously described [25] and by next-generation sequencing (NGS) (Miseq, Illumina, San Diego, CA, USA). cDNA libraries for NGS were prepared by using a NEBNext Ultra RNA Library Prep Kit for Illumina (NEB, Ipswich, MA, USA). The libraries prepared were sequenced by using a Miseq Reagent Kit V2 (Illumina), and the fragments obtained were mapped to the reference sequences of influenza A viruses by using Genomics Workbench software (CLC Bio, Aarhus, Denmark). The sequences obtained were used as query sequences in BLAST searches, then the genetic origins of each segment were determined based on the genetic lineages of the strains with the highest identity of the known lineage, such as A(H1N1)pdm09, North American Triple reassortant, and human seasonal lineages and so on. The nucleotide sequences obtained in the present study have been deposited in GISAID Epiful database (http://www.gisaid.org), strain IDs are available on Additional file 1.

### Statistical analysis

The age and production stage of the pigs from which the nasal swabs were collected were recorded during the visit. In addition, epidemiologic data (including farm type, number of pigs, availability of disinfection facilities, other animals on farm, operation type, the type of pig house, introduction of pigs from other farms, vaccine history, and so on; Additional file 2) were collected as variables for statistical analysis by interviewing the farmers on each farm. The variables were defined as follows: farm type (“corporate farm” managed by a company or “family-operated” farm managed by owner’s family); numbers of pigs (numbers of sows, suckling pigs, weanling pigs, fattening pigs, and boars); disinfection facility (availability of shower or tank for humans or vehicles at the farm entrance); other animals on farm (poultry, cats, dogs, cattle, etc. kept or not kept); operation type (farrow-to-finish or wean-to-finish or farrow-to-wean, or a combination of these three operation types); type of pig house (windowless or open-sided or semi-open-sided; open- and semi-open-sided pig houses allow wildlife such as birds to enter easily); introduction of pigs from other farms (pigs for breeding purchased or produced on the premises); vaccine history (types of vaccines used on farms, e.g., classic swine fever, foot-and-mouth disease, porcine reproductive and respiratory syndrome, Aujeszky’s disease, *Mycoplasma* spp.); respiratory disease histories in pigs or working staff (respiratory symptoms observed in pigs or working staff within the 6 months before our sampling); and presence of livestock farms within 100 m.

Influenza positivity was confirmed through virus isolation. The age and stage distributions of the pigs investigated were assessed by Fisher’s exact test to identify the age or production group most susceptible to subclinical infection with IAV-S. A farm was considered positive when at least one virus was isolated from the nasal swabs tested. Differences in management procedures between influenza-positive and influenza-negative farms were assessed by using a logistic regression model in univariate analysis. IAV-S positivity or negativity was set as the dependent variable, and all variables describing farm characteristics were evaluated individually in regression models; selected variables were evaluated simultaneously in multivariate analysis. Independent variables were identified by calculating Pearson’s correlation coefficient, then variables with a high correlation coefficient (>0.6) were excluded to avoid multicollinearity. Finally, variables among influenza-positive farms were analyzed to reveal whether risk factors for subclinical infection with IAV-S differed among strains. All of the statistical procedures were performed in STATA SE version 12 (LightStone, Tokyo, Japan).

## Results

The 250 farms analyzed comprised 45 corporate farms, 189 family-operated farms (including smallholders), and 16 farms for which no information regarding farm type was obtained. The largest farm had 82,700 pigs, and the smallest farm had 6 pigs (median, 72); 2 farms did not report the number of pigs. Among the 250 farms were 133 farrow-to-finish farms that maintained sows and fed the offspring until they reached market weight, 30 wean-to-finish farms that purchased weanling piglets and raised them to finishing, and 8 farrow-to-wean farms from which piglets were sold after they were weaned; the remaining 79 farms used a combination of these three operation types. Vaccines against swine influenza were not available in Vietnam; the most common vaccines used at the farms were for classic swine fever (75.6 % of the farms investigated), foot-and-mouth disease (52.0 %), and porcine reproductive and respiratory syndrome (29.2 %). Dogs and poultry (chickens or ducks) were the other animals most commonly kept on pig farms (60.4 %). In addition, 27 farms reported respiratory symptoms in pigs, represented by coughing and nasal discharge, within the 6 months before our sampling; 5 of these 27 farms reported concurrent respiratory symptoms in working staff.

The number of pigs slaughtered daily ranged from 70 to 900 (median, 117.5) at the 10 slaughterhouses investigated. Finishing pigs were slaughtered within 6 to 48 h after arriving at a slaughterhouse from a pig farm. Of the 10 slaughterhouses, 8 processed pigs only; both pigs and cattle were killed at the remaining 2 slaughterhouses.

A total of 172 IAV-S—43 A(H1N1)pdm09, 47 H1N2, and 82 H3N2 subtype—were isolated from the 6659 nasal swabs collected from clinically healthy pigs at pig farms (Table 1). No viruses were isolated from the 375 nasal swabs collected at slaughterhouses. Virus isolation rates differed significantly between production stages (Fisher’s exact test: *P* < 0.001): 62.2 % of the viruses were isolated from weanling pigs compared with 37.8 % from fattening pigs (Table 1). Pigs from which IAV-S were isolated ranged in age from 3 weeks to 4.5 months (median, 1.5 months); no viruses were isolated from suckling pigs younger than 3 weeks or older than 6 months, including finishing pigs, sows, and boars.

**Table 1:** Specimen collection and IAV-S isolated from different pig production stages and age groups in Vietnam

		IAV-S positive		IAV-S negative		*P* value^a^	Number of IAV-S in each subtype		
		number	%	number	%		H1N1	H1N2	H3N2
Stage	Suckling pig	0	0	290	4.2	<0.001	0	0	0
	Weanling pig	107	62.2	3268	47.6		19	17	71
	Fattening pig	65	37.8	2671	38.9		23	32	10
	Finishing pig^b^	0	0	375	5.5		0	0	0
	Sow	0	0	240	3.5		0	0	0
	Boar	0	0	18	0.3		0	0	0
Age	0 to <1 month old	22	12.8	467	6.8	<0.001	0	7	15
	1 to <2 months old	80	46.5	2593	37.8		17	12	51
	2 to <3 months old	46	26.7	2030	29.6		14	20	12
	3 to <6 months old	24	14.0	1139	16.6		11	10	3
	>6 months old	0	0	633	9.2		0	0	0
Number of viruses isolated		172					42	49	81

^a^Fisher’s exact test

^b^Samples from finishing pigs were collected at slaughterhouses

At least one IAV-S was isolated from each of 22 of the 250 farms tested, including 16 corporate farms housing 1392 to 82,700 pigs (median, 4205) and 6 family-operated farms with 20 to 654 pigs (median, 45). Univariate analysis regarding farm management practices between the 22 IAV-S-positive farms and the 228 IAV-S-negative farms revealed that the factors of farm type, total number of pigs, availability of a shower-in facility for humans, presence of a disinfection facility for vehicles, and presence of other animals on the farm were significantly associated with IAV-S subclinical infection (Table 2). The likelihood of IAV-S positivity was significantly increased when a farm was a corporate rather than a family-owned operation (*P* < 0.001) and when it had more than 1000 pigs compared with fewer than 50 pigs (*P* < 0.001). In addition, the availability of disinfection facilities for either humans or vehicles at the farm entrance increased the likelihood of IAV-S subclinical infection (*P* < 0.001). The likelihood of IAV-S positivity was also significantly increased in farms where disease histories in pigs (*P* = 0.002) and staffs (*P* = 0.032) had been observed within 6 months before sampling (Table 2). In contrast, pig farms that also had poultry (chicken, duck), dogs, cats, cattle, or other animals had a significantly lower odds ratio than did the farms that reared pigs only (*P* = 0.005). No correlations were found between the likelihood of IAV-S subclinical infection and presence of pig and/or other livestock farms within 100 m.

**Table 2:** Differences in farm characteristics between IAV-S-positive and IAV-S-negative farms

Variable		IAV-S-positive farms,		IAV-S-negative farms,		Odds ratio	95 % CI	*p* value^a^
		no.	(%)	no.	(%)			
Farm type^b^	Family-operated farm	6	(27.3 %)	183	(86.3 %)		reference	
	Corporate farm	16	(72.7 %)	29	(13.7 %)	16.83	6.09 to 46.52	<0.001
Total number of pigs	<50	4	(18.2 %)	108	(47.8 %)		reference	
	50 to 500	1	(4.5 %)	83	(36.7 %)	0.33	0.04 to 2.97	0.319
	501 to 1000	1	(4.5 %)	14	(6.2 %)	1.93	0.20 to 18.50	0.569
	≧1000	16	(72.7 %)	21	(9.3 %)	20.57	6.25 to 67.70	<0.001
Shower-in facility for humans^c^	No	5	(26.3 %)	195	(85.9 %)	17.06	reference	
	Yes	14	(73.7 %)	32	(14.1 %)		5.75 to 50.62	<0.001
Disinfection facility for vehicles^c^	No	7	(31.8 %)	194	(85.8 %)	12.99	reference	
	Yes	15	(68.2 %)	32	(14.2 %)		4.92 to 34.34	<0.001
Other animals on farm^d^	No	9	(42.9 %)	33	(16.1 %)	0.26	reference	
	Yes	12	(57.1 %)	172	(83.9 %)		0.10 to 0.66	0.005
Type of operation	Not farrow-to-finish^e^	5	(22.7 %)	91	(39.9 %)		reference	
	Including farrow-to-finish	17	(77.3 %)	137	(60.1 %)	2.26	0.81 to 6.34	0.12
Type of pig house^f^	Open- or semi-open-sided house	20	(90.9 %)	178	(78.1 %)	0.36	reference	
	Windowless	2	(9.1 %)	50	(21.9 %)		0.08 to 1.58	0.173
Introduction of pigs	No	1	(5.6 %)	30	(19.7 %)	4.18	reference	
	Yes	17	(94.4 %)	122	(80.3 %)		0.54 to 32.67	0.173
Vaccination^g^	No	1	(5.3 %)	32	(15.1 %)	3.20	reference	
	Yes	18	(94.7 %)	180	(84.9 %)		0.41 to 24.82	0.266
Respiratory diseases history in pig^h^	No	15	(68.2 %)	208	(91.2 %)		reference	
	Yes	7	(31.8 %)	20	(8.8 %)	4.85	1.77 to 13.29	0.002
Respiratory diseases history in working staff^h^	No	20	(90.9 %)	225	(98.7 %)		reference	
	Yes	2	(9.1 %)	3	(1.3 %)	7.50	1.18 to 47.54	0.032
Livestock farm	No	14	(68.2 %)	116	(91.2 %)		reference	
within 100 m^i^	Yes	6	(31.8 %)	111	(8.8 %)	0.45	0.17 to 1.21	0.112
Pig farm within 100 m	No	14	(68.2 %)	157	(91.2 %)	0.96	reference	
	Yes	6	(31.8 %)	70	(8.8 %)		0.35 to 2.61	0.938

^a^Logistic regression analyses were performed to calculate odds ratio of IAV-S-positive vs -negative farms for each variable

^b^Corporate farm managed by a company, and family-operated farm managed by owner’s family

^c^Disinfection facilities for humans or vehicles at the farm entrance

^d^Poultry (chickens, ducks), dogs, cats, cattle, etc

^e^Farrow-to-wean or wean-to-finish farms

^f^Windowless house or (semi-) open-sided house

^g^Vaccines for diseases other than swine influenza. Swine influenza vaccine is not available in Vietnam

^h^Respiratory symptoms observed in pigs or working staff within the 6 months before our sampling

^i^Presence of livestock farms including pig farm within 100 m

The subsequent regression multivariate analysis showed that the factor “total number of pigs” on the pig farm was significantly associated with subclinical infection with IAV-S (Table 3). Specifically, the likelihood of IAV-S positivity was 25.54 times higher for farms with 1000 pigs or more than for those with fewer than 1000 pigs (*P* < 0.001) (Table 3). The four variables included in the regression analysis—“type of operation”, “total number of pigs”, “type of pig house”, and “other animals on farm”—were selected on the basis of the Pearson correlation analysis (see Additional file 3). Because “total number of pigs” was highly associated with “corporate farms”, “shower-in facility for humans”, and “disinfection facility for vehicles”, those three variables were excluded from the multivariate model. In addition the presence of other animals was negatively associated with the total number of pigs (Additional file 3). We therefore could not rule out the possibility that the presence of other animals were confounded with the total number of pigs. However, because “other animals on farm” had been considered as a risk factor for IAV-S infection in other previous study [16], we used this variate in the multivariate analysis. As the result, it did not turn out that it has effect on the IAV-S positivity in our multivariate analysis (Table 3).

**Table 3:** Logistic estimates for IAV-S positivity according to selected variables

Variable		*P*	Odds ratio	95 % CI
Total number of pigs	<1000		reference	
	≧1000	<0.001	25.54	7.42 to 87.98
Type of operation	Not farrow-to-finish		reference	
	Including farrow-to-finish	0.85	0.89	0.24 to 3.24
Type of pig house	Open-sided or semi-open-sided house		reference	
	Windowless	0.26	0.36	0.06 to 2.10
Other animals on farm	No		reference	
	Yes	0.55	0.70	0.21 to 2.29
Constant		<0.001	0.13	

**Fig. 1:**
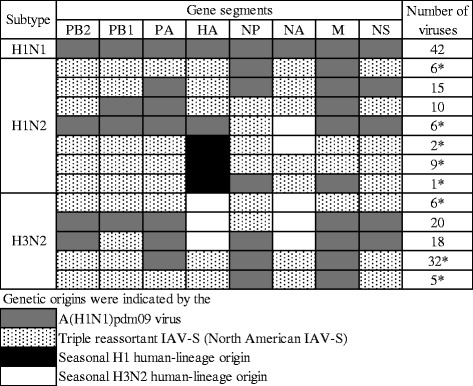
Gene constellations of the IAV-S identified. Gene segments derived from A(H1N1)pdm09 viruses are represented as gray squares; those from enzootic strains are represented as stippled (North American triple-reassortant IAV-S), black (human, seasonal, H1 origin), and white (human, seasonal, H3 origin) squares. PB2, polymerase gene 2; PB1, polymerase gene 1; PA, polymerase gene A; HA, hemagglutinin gene; NP, nucleoprotein gene; NA, neuraminidase gene; M, matrix gene; and NS, non-structural gene. The numbers of the isolates in each genotype are indicated in the rightmost column. Asterisks indicate the novel genotypes found in this study

All of the genetic origins of each segment of the isolates in this study showed more than 97 % identities with strains whose genetic origins had been defined in previous studies. All of the segments of the H1N1 isolates were derived from A(H1N1)pdm09 viruses (Fig. 1). In contrast, the H1N2 and H3N2 IAV-S isolated in this study were reassortants that had at least two genes derived from enzootic strains in the Vietnamese pig population (Fig. 1) [24, 29]. In particular, 113 IAV-S (87 %) out of 130 H1N2 and H3N2 isolates were reassortants between endemic strains and A(H1N1)pdm09 viruses (Fig. 1). To examine whether the risk factors for IAV-S subclinical infection differed among strains, we compared management practices between A(H1N1)pdm09-positive farms and farms positive for endemic IAV-S. One corporate farm where both A(H1N1)pdm09 virus and an enzootic strain were isolated was included as being both A(H1N1)pdm09v-positive and enzootic-positive (Table 4). This comparison revealed that two variables—“total number of pigs” and “other animals on farm”—differed significantly between the two kinds of farms (Fisher’s exact test: *P* < 0.01) (Table 4). That is, farms with 1,000 pigs or more had a significantly higher risk of infection by endemic strains infection than did the farms where A(H1N1)pdm09 viruses were isolated. No enzootic strains were isolated from family-operated farms with fewer than 50 pigs, whereas A(H1N1)pdm09 viruses were isolated from 4 family-operated farms. All 8 farms from which A(H1N1)pdm09 viruses were isolated were rearing other animals in addition to pigs. In contrast, the 9 farms, out of 15 farms positive for enzootic IAV-S, were raising only pigs.

**Table 4:** Differences in farm characteristics between A(H1N1)pdm09v-positive and enzootic IAV-S-positive farms^a^

Variable		A(H1N1)pdm09v-positive farms, no. (%)	Enzootic IAV-S-positive farms, no. (%)	*P* value^*^
Total number of pigs	<50	4 (50.0 %)	0 (0.0 %)	
	50 to 500	0 (0.0 %)	1 (6.7 %)	
	500 to 1000	0 (0.0 %)	1 (6.7 %)	
	>1000	4 (50.0 %)	13 (86.7 %)	0.01
Other animals on farm	No	0 (0.0 %)	9 (60.0 %)	
	Yes	8 (100.0 %)	5 (33.3 %)	
	No answer	0 (0.0 %)	1(6.7 %)	0.01
Farm type	Family-operated farm	4 (50.0 %)	2 (13.3 %)	
	Corporate farm	4 (50.0 %)	13 (86.7 %)	0.13
Shower-in facility for humans	No	3 (37.5 %)	2 (13.3 %)	
	Yes	5 (62.5 %)	10 (66.7 %)	
	No answer	0 (0.0 %)	3 (20.0 %)	0.24
Disinfection facility for vehicles	No	3 (37.5 %)	4 (26.7 %)	
	Yes	5 (62.5 %)	11 (73.3 %)	0.66
Type of operation	Not farrow-to-finish	3 (37.5 %)	2 (13.3 %)	
	Including farrow-to-finish	5 (62.5 %)	13 (86.7 %)	0.30
Type of pig house	Open-sided or semi-open-sided house	8 (100.0 %)	13 (86.7 %)	
	Windowless	0 (0.0 %)	2 (13.3 %)	0.53
Introduction of pigs from external source	No	0 (0.0 %)	1 (6.7 %)	
	Yes	7 (87.5 %)	11 (73.3 %)	
	No information	1 (12.5 %)	3 (20.0 %)	1.00
Vaccination	No	0 (0.0 %)	1 (6.7 %)	
	Yes	5 (62.5 %)	14 (93.3 %)	
	No answer	3 (37.5 %)	0 (0.0 %)	0.03

^*^Fisher’s exact test was applied to compare proportions between A(H1N1)pdm09v positive- and enzootic IAV-S-positive- farms

^a^One farm from which both A(H1N1)pdm09 virus and an enzootic strain were isolated was included as being both A(H1N1)pdm09v-positive and enzootic-positive

## Discussion

To date, few studies [30–32] have addressed the subclinical forms of IAV-S, because the presence of IAV-S on a farm has been demonstrated mainly through passive or serologic surveillance efforts. Our active virologic monitoring indicates that IAV-S have spread subclinically in the Vietnamese pig population. One important finding of our study is that, in Vietnam, IAV-S are more prevalent on farms with 1000 pigs or more than on smaller farms. Modern industrialized farms have emerged in Vietnam since 2000 and have gradually replaced the traditional, small, family-run farms [21, 23], as has occurred in the United States and in European countries over the last several decades [31, 33]. Rearing a large number of pigs on a farm is recognized as one of the most influential risk factors for IAV-S seropositivity in sows and fattening pigs [1, 4, 12, 17]. Our current results coincide with these previous findings, although the earlier serologic studies did not differentiate between subclinical infections and typical, clinical infections in pigs [1, 4, 12, 17]. Housing large numbers of susceptible pigs closely together likely provides appropriate conditions for IAV-S to circulate continuously in the pig population on a farm [12, 31]. In addition, the intensification of husbandry practices associated with large-scale farms might increase the frequency of movement of workers and vehicles to and within a farm, thus increasing the risk of pathogen transmission [34].

We isolated A(H1N1)pdm09 viruses from both corporate and family-run farms. This result is consistent with other reports, such as those of the large, corporate farms in the United States [26] and Canada [35], as well as small family-operated farms in Peru [36] and Canada [37]. According to the genetic analysis, Vietnamese H1N1 isolates in this study showed the high identity with A(H1N1)pdm09 viruses, reflecting viral transmission from humans to pigs, as has been seen in many other countries [35, 38–40]. Such human-to-pig transmission of A(H1N1)pdm09 viruses increases global public health concerns, because reassortment with enzootic IAV-S could generate influenza A viruses with novel gene combinations in the pig population. Pigs are highly susceptible to A(H1N1)pdm09 viruses [41], and our data suggest that efforts to prevent the introduction of these strains into farms have not been effective. According to the NCBI Influenza Virus Resource, A(H1N1)pdm09 viruses had been isolated from pigs in 34 countries as of 2015 [42]. Unsurprisingly, many novel reassortants between A(H1N1)pdm09 viruses and the enzootic IAV-S have arisen in many countries and regions [43–49]. One such example with public health implications is a novel reassortant H3N2v, which is a triple-reassortant H3N2 IAV-S that contains an M gene from an A(H1N1)pdm09 virus. The H3N2v isolate was first identified from human in the United States in July 2011, and more than 300 human cases have been reported, although human-to-human transmission is limited [50].

According to the results of BLAST searches, most of the H1N2 and H3N2 IAV-S that we isolated were reassortants between enzootic strains in Vietnamese pigs and A(H1N1)pdm09 viruses. In particular, 97 % of these reassortants (125 isolates) were isolated from farms with 1,000 pigs or more. To our knowledge, eight out of 12 genotypes of H1N2 and H3N2 subtypes are never reported outside Vietnam (Fig. 1) [44, 51–55]. These findings suggests that large-scale farms provide an environment that is more conducive than that of small-scale farms for IAV-S to exchange their gene segments with each other and with other viruses. Subclinical infections likely spread more slowly than does the epidemic form that leads to acute respiratory disease on a farm [11, 12]. If so, subclinical infections with IAV-S warrant increased attention to prevent the generation of novel viruses, because prolonged virus circulation would increase the opportunity for reassortment events. We recently reported that numerous IAV-S with various gene constellations arose in weanling pigs co-infected with H1N1 and H3N2 IAV-S on a large, corporate farm [56]. In particular, plaque purification assays of nasal swabs revealed that at least 16 different constellations of gene segments emerged within a single co-infected pig that lacked clinical signs.

Young pigs of the weaning and early-fattening stages appear to play an important role in the interepizootic survival of IAV-S. High virus-isolation rates from clinically healthy pigs have been obtained from those aged 4 to 12 weeks in Thailand [7, 47], 12 weeks in Japan [57], and 4 to 12 weeks in the current study. These ages coincide with the waning of maternal antibodies in piglets; maternal antibodies against IAV-S gradually decline through 10 weeks of age [11]. Continuous production of susceptible piglets on large-scale farms might provide a chain of naïve hosts to maintain subclinical infection with IAV-S. In fact, recurrent IAV-S infections in young pigs on large commercial farms have been reported. For example, on a 300-sow farrow-to-finish swine farm in Spain, a specific IAV-S repeatedly affected a group of pigs over the course of 6 months, and this particular strain was the isolate most frequently found when the pigs were tested as 3- and 7-week-olds [10]. Interestingly, three of the pigs examined were infected with the same subtype strain twice on two different occasions during the 6-month period. Our previous active surveillance in Thailand alternately isolated H1N1 and H3N2 IAV-S from weanling pigs (age 4 to 8 weeks) at a 17-month interval [7]. In addition to young pigs, sows appear to be frequently infected with IAV-S, perhaps because they typically are the only group with any longevity (usually 4 to 6 years) on a farm; the likelihood of infection with a pathogen increases with time [15]. Our previous study and those of others have demonstrated an increased seropositivity rate against IAV-S in sows at farrow-to-finish farms [7, 12, 15, 58]. However, we did not isolate any IAV-S from clinically healthy sows in our current study or during our previous active surveillance in Thailand and Vietnam [7, 24, 47]. Therefore, how sows are involved in asymptomatic circulation of IAV-S on pig farms remains unclear.

Subclinical infection with IAV-S is currently not considered to have a pronounced negative effect on growth performance on pig farms. However, Er et al. [59] recently demonstrated that subclinical infection of pigs on Norwegian farms with A(H1N1)pdm09 viruses reduced both feed conversion efficiency and average daily growth. Interestingly, the adverse effect lasted longer than the viral shedding period (about 7 days). Specifically, pigs infected when they weighed 33 to 60 kg required 8.0 kg more feed to reach 100 kg bodyweight than did unexposed pigs. Consequently, the infected pigs took longer to reach market weight [55]. Although our current investigation did not directly address such adverse effects of subclinical infection in Vietnamese pigs, similar effects might be presumed.

In contrast to the situation on farms from which we isolated enzootic strains, the farms that harbored A(H1N1)pdm09 viruses were more likely to have kept animals other than pigs. Having mammalian pets on a farm was a risk factor for IAV-S seropositivity on Malaysian pig farms [16]. However, whether these other animals serve as “couriers” for spreading influenza A viruses within a farm is unclear, although A(H1N1)pdm09 viruses have a wide host range among mammals [41], including dogs [60] and cats [61]. In addition, we could not rule out the possibility that ‘presence of other animals’ is confounded with the number of pigs as concerned in the univariate analysis. Further study would be needed to understand a role of the presence of other animals in pig farms.

Introduction of pigs from outside a farm is another important risk factor for seropositivity against IAV-S in pigs [5, 12, 15, 16]. Many outbreaks of swine influenza are clearly related to the introduction of infected pigs [62]. However, our analysis failed to reveal a significant relationship between subclinical infection with IAV-S and the introduction of pigs, although 17 of the 22 IAV-S-positive farms had obtained gilts or weanling pigs from external sources. This discrepancy may be explained in part by the fact that 32 % (80 farms) of the farms had not recorded their purchase histories; these missing values consequently were excluded from our statistical analysis. Evaluating the effect of the introduction of pigs on subclinical infection with IAV-S on pig farms merits increased attention. Interestingly, 90 % of IAV-S positive farms (20 farms) had open- or semi-open-sided houses although it did not show significant P value compared with the positive farms having windowless pig houses, suggesting that the type of pig house may be worthy of attention in future studies. Although involvement of seasonality, presence of other respiratory pathogens, and humidity or temperature of barns, that were considered as risk factors for the IAV-S occurrence by previous studies [1, 4, 62], was unable to be evaluated in this study, those could be risk factors even in subclinical infections of IAV-S in pig farms and further study on those variables would provide useful information.

## Conclusions

Taken together, our findings demonstrate the asymptomatic circulation of IAV-S in the Vietnamese pig population. Raising a large number of pigs on a farm has the strongest impact on the incidence of subclinical IAV-S infection, although not all the large-scale farms we evaluated suffered from subclinical IAV-S infection. In fact, 29 of the 37 large, corporate farms in our surveillance yielded no IAV-S, raising the possibility that risk factor(s) for subclinical infection other than a large number of pigs exist within this type of farm. Further active surveillance to define risk factors for subclinical infection with IAV-S in Vietnam would provide early detection of a virus with pandemic potential and would also mitigate economic losses in the pig industry.

## Additional files

Additional file 1: Isolate_IDs and virus names deposited in GISAID Epiful database. (XLS 48 kb)
Additional file 2: Farm and sample information sheets used in the interviews with farm owners in this study. (DOC 107 kb)
Additional file 3: Pearson correlation matrix for the variables investigated. Pearson’s correlation coefficient was used to identify independent variables collected in this study. (XLS 45 kb)
