# Supplementary material for: Effect of herd size on subclinical infection of swine in Vietnam with influenza A viruses
Source: BMC Vet Res. 2016 Oct 10;12:227. doi: 10.1186/s12917-016-0844-z (PMC5057248; doi:10.1186/s12917-016-0844-z)
Supplement: Additional file 2: — Farm and sample information sheets used in the interviews with farm owners in this study. (DOC 107 kb) [file 12917_2016_844_MOESM2_ESM.doc]

FARM INFORMATION SHEET

Farm ID : ………………. Farm name: ………………………………………………… Date:………………

Address: Province:……………District………………Commune:……………Village:……………..Street:………

GPS:………………………………………..

| No | Questions | Answer | | | | | | | | | | | | | | |
| --- | --- | --- | --- | --- | --- | --- | --- | --- | --- | --- | --- | --- | --- | --- | --- | --- |
| Q1 | Farm type | Corporate farm  □ | | | | | | Family-operated farm  □ | | | | | | | | |
| Q2 | Type of pig house  (Windowless house or (semi-) open-sided house.) | Open-sided house  □ | | | Windowless house  □ | | | | | Semi-open-sided house  □ | | | | | | |
| Q3 | Type of operation | Farrow to finish  □ | | | Farrow to weaning  □ | | | | | Weaning to finish  □ | | | | | | |
| Q4 | Pig stage |  | | | Age | | | | | Remarks | | | | | | |
|  |  | Suckling pig* | | | 1－..……days old | | | | | *Piglet with Sow | | | | | | |
|  |  | Weaner** | | | …………－…….…..  days / months old | | | | | **Piglets separated from Sow | | | | | | |
|  |  | Fattening*** | | | …………－…….…..  days / months old | | | | | ***Pigs in fattening stage | | | | | | |
| Q5 | Number of pigs (heads) in farm. | Sow | | | Suckling pig | | | Weanling pigs | | | | | Fattening pigs | | | Boar |
|  |  |  |  |  |  |  |  |  |  |  |  |  |  |  |  |  |
| Q6 Introduction of pigs   |  | Sow (gilt) | Boar | Weanling pigs | Other  ( ) | | --- | --- | --- | --- | --- | | Where?  (Farm or Company name, Village/District/Province) |  |  |  |  | | Age (week-old) |  |  |  |  | | How often? | □: Continuously  □: Not often | □: Continuously  □: Not often | □: Continuously  □: Not often | □: Continuously  □: Not often | | If ‘Continuously’, provide average number of pigs introduced. | …………….pigs  in a week/month/year | …………….pigs  in a week/month/year | …………….pigs  in a week/month/year | …………….pigs  in a week/month/year | | If ‘not often’, provide number of pigs and date you introduced pigs last time. | …………….pigs  Year ………….  Month ……….. | …………….pigs  Year ………….  Month ……….. | …………….pigs  Year ………….  Month ……….. | …………….pigs  Year ………….  Month ……….. | | | | | | | | | | | | | | | | | |
|  | | | | | | | | | | | | | | | | |
| Q7 | Vaccination | |  | Sow | Boar | Weanling and fattening pigs | | --- | --- | --- | --- | | FMD |  |  |  | | HCV |  |  |  | | PRRS |  |  |  | | Swine Influenza |  |  |  | | Aujeszky’s disease |  |  |  | | Mycoplasma |  |  |  | | Other (………….) |  |  |  | | Other (………….) |  |  |  | | | | | | | | | | | | | | | |
| Q8 | Other animals on farm |  | Duck | Chicken | | Cattle | | | Buffalo | | Goat | | | Dog | Other  : ……….. | |
|  |  | Number |  |  | | |  | |  | | |  | |  |  | |
| Q9 | Respiratory diseases history in pig and working staff within 6 months | + In pig: 　　□No □Yes  When:……………………………...  Clinical sign: …………………………………………..…….  Name of disease: ……………………………………….…………….......  + In staff: 　　□No □Yes  When:…………………………….  Clinical sign: ……………………………………..………….  Name of disease: ……………………………………….……………....... | | | | | | | | | | | | | | |
| Q10 | Disinfection facilities for vehicle or human | □No □Yes　(for vehicle) □Yes　(Shower-in for human) | | | | | | | | | | | | | | |
| Q11 | Other livestock farms  within 100m | □No □Yes Farm (Pig, chicken…): …………………………………… | | | | | | | | | | | | | | |
| Q13 | Memo | | | | | | | | | | | | | | | |

Farm ID : ……………….

SAMPLE INFORMATION SHEET

Farm ID: ………………Farm name: ……………………………………… Date:………………………

Operator name: ………………………………….Operator phone:………………………………………..

*Remark*: Type of pig:

|  | *Identified No.* |  |
| --- | --- | --- |
| *Weanling pig* | *1* | *Piglet weaned from sow* |
| *Fattening pig* | *2* | *Pigs in fattening stage* |
| *Suckling pig* | *3* | *Piglet with sow* |
| *Sow* | *4* |  |
| *Boar* | *5* |  |

| No | Sample ID | Pig ID | Type of pig | Age  weeks/months | Sexuality  Male/Female | When was the pig introduced? | Symptoms  (If any) |  |
| --- | --- | --- | --- | --- | --- | --- | --- | --- |
| 1 |  |  |  |  |  |  |  |  |
| 2 |  |  |  |  |  |  |  |  |
| 3 |  |  |  |  |  |  |  |  |
| 4 |  |  |  |  |  |  |  |  |
| 5 |  |  |  |  |  |  |  |  |
| 6 |  |  |  |  |  |  |  |  |
| 7 |  |  |  |  |  |  |  |  |
| 8 |  |  |  |  |  |  |  |  |
| 9 |  |  |  |  |  |  |  |  |
| 10 |  |  |  |  |  |  |  |  |
| 11 |  |  |  |  |  |  |  |  |
| 12 |  |  |  |  |  |  |  |  |
| 13 |  |  |  |  |  |  |  |  |
| 14 |  |  |  |  |  |  |  |  |
| 15 |  |  |  |  |  |  |  |  |
| 16 |  |  |  |  |  |  |  |  |
| 17 |  |  |  |  |  |  |  |  |
| 18 |  |  |  |  |  |  |  |  |
| 19 |  |  |  |  |  |  |  |  |
| 20 |  |  |  |  |  |  |  |  |
| 21 |  |  |  |  |  |  |  |  |
| 22 |  |  |  |  |  |  |  |  |
| 23 |  |  |  |  |  |  |  |  |
| 24 |  |  |  |  |  |  |  |  |
| 25 |  |  |  |  |  |  |  |  |
| 26 |  |  |  |  |  |  |  |  |
| 27 |  |  |  |  |  |  |  |
| 28 |  |  |  |  |  |  |  |
| 29 |  |  |  |  |  |  |  |
| 30 |  |  |  |  |  |  |  |

Farm ID: ………………Farm name: ……………………………… Date:………………………
